# Typhoidal tularemia with pancytopenia and pericarditis in a georgian adolescent

**DOI:** 10.1016/j.idcr.2025.e02451

**Published:** 2025-12-07

**Authors:** Marika Tsereteli, David Chakhunashvili, Nino Abuladze, Tamta Matchavariani, Giorgi Chakhunashvili, David Tsereteli, Mariam Kakabadze, Paata Imnadze

**Affiliations:** a National Center for Disease Control and Public Health (NCDC&PH), Tbilisi, Georgia; b Tbilisi State Medical University and Ingorokva High Medical Technology University Clinic, Tbilisi, Georgia; c Tbilisi State Medical University, Tbilisi, Georgia

**Keywords:** Typhoidal Tularemia, *Francisella tularensis*, Pericarditis, Pancytopenia, Serology

## Abstract

We report a rare case of typhoidal tularemia in an 18-year-old male from Kvemo Kartli, southern Georgia. The patient presented with a two-week history of high-grade fever, severe malaise, anorexia, and laboratory evidence of pancytopenia with hypoglycemia. The clinical course was complicated by pericarditis. Hematologic malignancy was initially suspected, but bone marrow aspiration revealed no blasts or dysplasia. Serologic testing confirmed *Francisella tularensis* infection. The patient responded to targeted antimicrobial therapy with full recovery. This case highlights the importance of considering tularemia in the differential diagnosis of febrile illnesses with cytopenia in endemic rural settings. Although tularemia is considered rare in many parts of the world, Georgia and neighboring regions have reported sporadic outbreaks, particularly in rural areas with close human–animal interactions. The typhoidal form is especially challenging to diagnose due to the absence of localized lesions, often mimicking other systemic febrile illnesses such as brucellosis, typhoid fever, or hematologic disorders. Early recognition is critical, as delayed treatment can result in severe complications, including multiorgan involvement, cardiovascular manifestations, and hematologic abnormalities. Reporting atypical presentations contributes to a better understanding of the disease spectrum and aids clinicians in endemic areas to consider tularemia even when classical signs are absent.

## Highlights

• Typhoidal tularemia may mimic hematologic malignancy due to pancytopenia.
• Pericarditis is a rare but possible complication of tularemia.
• Bone marrow aspiration helps exclude malignancy while also directing attention to potential infectious causes.
• Serology remains a valuable diagnostic tool in resource-limited settings.

## Introduction

Tularemia is a zoonotic infection caused by *Francisella tularensis*, a highly infectious, facultative intracellular gram-negative bacterium. Transmission occurs through arthropod bites, direct animal contact, inhalation, or contaminated food and water [1]. Clinical presentations vary, with ulceroglandular tularemia being the most common. The typhoidal form, representing 10–15 % of cases, is characterized by systemic illness without localizing signs, leading to diagnostic challenges [1], [2], [3]. Comprehensive epidemiologic and clinical overviews have been published in recent reviews [4], [5], and the World Health Organization highlights its ongoing public health importance [6]. In Eastern Europe, including Georgia, tularemia remains endemic, with periodic outbreaks reported [7], [8]. Hematologic abnormalities in tularemia are usually mild; severe bone marrow suppression with pancytopenia is rare [9], [10], [11]. Likewise, pericarditis is an unusual but potentially severe complication [12], [13], [14]. We describe a case of systemic tularemia in an adolescent male, complicated by pancytopenia and pericarditis.

## Case presentation

An 18-year-old male from a rural community in the Kvemo Kartli region of southern Georgia presented with a two-week history of fever (39°C), severe malaise, and anorexia. On admission, he was alert but profoundly fatigued. The patient had no prior significant medical history and no recent travel outside the region. He reported frequent contact with farm animals, including cattle and rabbits, occasional hunting activity in nearby forests and consumption of game meat. No history of tick bites was noted, and there was no known exposure to contaminated water sources. He had not taken any medication three months prior to hospitalization. On physical examination, mild hepatosplenomegaly was noted, although no skin lesions or ulcers were observed. Vital signs revealed bradycardia (heart rate 50 bpm) and mild hypotension (BP 100/65 mmHg). Given the combination of pancytopenia and systemic symptoms, an extensive infectious workup was initiated. Blood cultures, viral serologies, and autoimmune panels were performed to exclude other potential etiologies. The presence of multiple enlarged lymph nodes with preserved architecture further supported an infectious rather than malignant process. Transthoracic echocardiography findings of pericardial effusion, though mild, prompted early cardiology consultation to monitor for potential progression.

Laboratory investigations at presentation:

• Leukopenia: 2.99 × 10⁹/L (reference range: 3.91–10.90)

• Neutropenia: 0.50 × 10⁹/L (reference range: 1.80–6.98)

• Thrombocytopenia: 108 × 10⁹/L (reference range: 166–308)

• CRP: 31 mg/L (reference range: ≤10)

• Hypoglycemia: 38 mg/dL (reference range: 70–110)

Peripheral smear revealed no blasts. Within 48 h, pancytopenia worsened: WBC 2.51 × 10⁹/L, neutrophils 0.27 × 10⁹/L, platelets 68 × 10⁹/L. Bone marrow aspiration showed preserved cellularity without blasts or dysplastic changes.

Imaging findings:

• Ultrasound of lymph nodes showed multiple enlarged lymph nodes with preserved architecture (cervical up to 32 mm, axillary 27 mm, inguinal 25 mm).

• Transthoracic echocardiography demonstrated separation of the pericardial layers during diastole. The maximal pericardial effusion measured 7 mm in the apical view adjacent to the right atrium, and up to 13 mm in the subcostal view adjacent to the right-sided cardiac chambers. Additional measurements revealed a separation of 13 mm at the right ventricular apical segment and 11 mm at the apex in the apical four-chamber view.

Microbiological and serologic findings:

• Blood cultures: negative

• Serology for *Francisella tularensis*: ELISA IgM: 40 (cutoff <15), ELISA IgG: 210 (cutoff <15), MAT titer: 1:256 (positive, cutoff <1:128). MAT titer was checked twice at a two-week interval to increase diagnostic reliability.

Empirical antibiotic was initiated on admission, but without any effect on fever. Upon serologic confirmation, treatment was continued with Streptomycin 1 g IM injection q12hr. Within 72 h the patient developed vertigo, requiring a switch to Ciprofloxacin 500 mg twice daily for 21 days (adjusted per local guidelines), Gentamicin could not be used due to shortage. The patient’s fever subsided within one week. Cardiologist was also included in the management team and non-steroidal anti-inflammatory drug (NSAID) was added to the treatment scheme. At the end of treatment, pericardial effusion and hematologic indices improved progressively, and repeat echocardiography at two weeks demonstrated positive dynamic. At one-month follow-up, the patient remained asymptomatic with normalized blood count and no pericardial effusion.

## Discussion

This case illustrates two rare systemic manifestations of tularemia: pancytopenia and pericarditis. Pancytopenia is hypothesized to result from bone marrow suppression due to systemic inflammation or direct bacterial interaction with hematopoietic cells, although only sporadic cases have been documented [9], [10], [11]. Similarly, pericarditis in tularemia has been reported infrequently and may mimic viral or autoimmune etiologies [12], [13], [14]. The pathophysiology of pancytopenia in tularemia is not fully understood, but several mechanisms have been proposed, including cytokine-mediated suppression of hematopoietic progenitor cells, transient bone marrow infiltration by inflammatory cells, and immune-mediated destruction of peripheral blood cells [10], [11]. Similarly, pericarditis in tularemia may result from direct bacterial invasion of the pericardium or secondary immune-mediated inflammation, emphasizing the need for cardiac monitoring in severe systemic infections [12], [13], [14]. Historical reports [14] and modern case descriptions [12], [13] confirm that while rare, pericarditis should be recognized as part of the tularemia spectrum. Case series from North America and Europe emphasize the variability of clinical presentations, with atypical systemic and cardiac forms often being overlooked [15], [16], [17]. Other atypical cases have been reported, underscoring diagnostic challenges [18], [19].

The diagnostic process was initially confounded by hematologic findings suggestive of malignancy, prompting bone marrow evaluation. The preserved marrow morphology, coupled with the epidemiologic context (rural residence in an endemic area), prompted consideration of zoonotic infections. Serology confirmed tularemia, and the patient improved with targeted therapy.

This case underscores the need for heightened clinical suspicion for tularemia in patients with prolonged fever, cytopenia, and epidemiological risk factors in endemic regions. Early serological testing is essential to prevent unnecessary invasive procedures and delays in treatment.

This case also highlights the importance of a multidisciplinary approach. Coordination between infectious disease specialists, hematologists, and cardiologists facilitated timely diagnosis, guided appropriate antibiotic selection, and ensured careful monitoring of hematologic and cardiac complications. Furthermore, it underscores the utility of serologic testing in resource-limited settings where advanced molecular diagnostics may not be readily available. Sharing such cases can improve regional diagnostic awareness and inform public health strategies for zoonotic disease surveillance.

Tularemia should be included in the differential diagnosis of febrile illnesses with hematologic abnormalities, particularly in endemic rural areas. Although rare, systemic complications such as pancytopenia and pericarditis may occur. Prompt recognition and treatment are critical for favorable outcomes.

## Ethical approval

This study was conducted in accordance with the principles of the Declaration of Helsinki. As this was a retrospective single case report that did not involve any intervention or identifiable personal information, institutional review board (IRB) approval was not required under the policy of our institution.

single case reports do not require formal IRB approval.

## Sources of funding for your research

No research funding used for this case report

## Funding

This research did not receive any specific grant from funding agencies in the public, commercial, or not-for-profit sectors.

## Author statement

Marika Tsereteli drafted the manuscript and supervised the further preparation process. Marika Tsereteli and Nino Abuladze managed the patient. Tamta Matchavariani conducted cardiological and radiologic assessment and visualization. David Chakhunashvili participated in the design and interpretation of the studies. All other authors contributed to clinical interpretation, literature review, or critical revision of the manuscript for important intellectual content. All authors have read and approved the final version of the manuscript and agree to be accountable for all aspects of the work.

## CRediT authorship contribution statement

**Marika Tsereteli:** Writing – review & editing, Writing – original draft. **Nino Abuladze:** Writing – review & editing, Resources. **David Chakhunashvili:** Writing – review & editing, Formal analysis, Data curation, Conceptualization. **Giorgi Chakhunashvili:** Writing – review & editing, Conceptualization. **Tamta Matchavariani:** Visualization, Resources. **Mariam Kakabadze:** Writing – review & editing, Resources. **David Tsereteli:** Writing – review & editing, Formal analysis, Data curation, Conceptualization. **Paata Imnadze:** Writing – review & editing, Supervision.

## Declaration of Competing Interest

The authors have no conflicts of interest.
